# Identification of Chemical Components in Three Types of Rose Essential Oils Based on Gas Chromatography-Mass Spectrometry (GC-MS) and Chemometric Methods

**DOI:** 10.3390/molecules30091974

**Published:** 2025-04-29

**Authors:** Min Xu, Jia Cai, Long Wang, Shunpeng Zhu, Yangxi Chen, Yuchen Chen, Jie Zhong, Jiaxin Li, Peng Hu, Qiang Ye

**Affiliations:** 1 State Key Laboratory of Southwestern Chinese Medicine Resources, Chengdu University of Traditional Chinese Medicine, Chengdu 611137, China; 18381083126@163.com (M.X.); caijia@stu.cdutcm.edu.cn (J.C.); 18523939143@163.com (L.W.); zhushunpeng@stu.cdutcm.edu.cn (S.Z.); a18081033128@163.com (Y.C.); chenyuchen@stu.cdutcm.edu.cn (Y.C.); 15208410804@163.com (J.Z.); 15881415754@163.com (J.L.); 2 School of Pharmacy, School of Modern Chinese Medicine Industry, Chengdu University of Traditional Chinese Medicine, Chengdu 611137, China; 3 School of Health Preservation and Rehabilitation, Chengdu University of Traditional Chinese Medicine, Chengdu 611137, China

**Keywords:** Jinbian rose, Kushui rose, Pingyin rose, gas chromatography-mass spectrometry (GC-MS), chemometric methods, differentiation of rose varieties

## Abstract

Currently, the main types of roses circulating in China include Jinbian Rose, Kushui Rose and Pingyin Rose. Each type of rose has slight differences in usage and efficacy. There are many varieties of roses, and the quality of rose essential oils varies greatly. Almost no research has systematically studied the essential oils of various roses. In this experiment, three types of roses (Jinbian Rose, Kushui Rose, and Pingyin Rose) were selected as research subjects based on their efficacy and variety in the market. Essential oils were extracted from the three types of roses using hydrodistillation. Gas chromatography-mass spectrometry (GC-MS) was used to qualitatively analyze the volatile substances in the essential oils of different varieties of roses. The three types of rose essential oils were identified and differentiated using chemometric methods (including HCA, PCA, PLS-DA, and OPLS-DA). On the one hand, based on the GC-MS analysis results, 40, 48, and 40 volatile components were detected in Jinbian Rose, Kushui Rose, and Pingyin Rose, respectively. The chemical compositions were primarily dominated by macromolecular compounds such as long-chain alkanes, organic acids, and esters. On the other hand, eight markers with significant identification values were identified to distinguish among the three types of roses. In conclusion, based on GC-MS analysis and chemometric methods, this experiment distinguishes and identifies three types of roses from the perspective of essential oil components for developing an effective strategy for the identification of rose varieties.

## 1. Introduction

Roses have been used in China’s medical and food fields for over 1300 years [1]. They possess the effects of promoting blood circulation, relieving depression, harmonizing blood, and alleviating pain, and are commonly used in clinical settings for conditions such as liver and stomach bloating, poor appetite, nausea, menstrual irregularities, and injuries from falls [2]. Roses are rich in essential oils, which have special pharmacological effects. Rose essential oil is considered the top among vegetable oils, and the extracted oil has a rich, fragrant, and elegant floral scent with a wide range of uses [3]. Known as “liquid gold”, rose essential oil has various benefits, including promoting blood circulation and removing blood stasis, moisturizing the skin, possessing anti-aging abilities, exhibiting anti-oxidant properties and anti-bacterial effects, functioning as a laxative and diuretic, and promoting sleep. Additionally, it holds high research and application value in health food, medicine, food additives, and cosmetics [3,4]. There is a diverse range of oil-bearing rose varieties globally, primarily found in countries such as Bulgaria, Turkey, Iran, Morocco, and India. The varieties commonly used for rose oil extraction internationally include Damascus rose (*Rosa* × *damascena Herrm*), White rose (*R.* × *alba* L.), French rose (*R. gallica* L.), and Cabbage rose (*R.* × *centifolia*) [5,6]. The most commonly used roses in China are the Jinbian Rose (JBR) from Yunnan Province, the Kushui Rose (KSR) from Gansu Province, and the Pingyin Rose (PYR) from Shandong Province. The JBR is mainly used for the development and production of food products, while the KSR and PYR are primarily used for clinical medicinal purposes [7,8,9]. The prices of the three types of roses also vary. The KSR is the most expensive, costing around CNY 60 per 500 g, while the JBR and PYR are priced at CNY 25 and 40, respectively. The essential oil extracted from KSR has been officially certified by the International Organization for Standardization (ISO) as early as 2013, and a clear definition of its essential oil components has been established [10,11]. On 1 November 2024, the PYR essential oil received a “new business card” in the form of the national standard GB/T 43954-2024 “Double-Layered Red Rose Essential Oil” [12]. This will enhance the quality level of products, expand the international market, and play an important role in improving the quality and safety reliability of rose essential oil products, as well as better meeting market demands [7].

At present, the clinical applications of JBR, KSR, and PYR are mainly reflected in promoting blood circulation and removing blood stasis, soothing the liver and relieving depression, regulating menstruation and relieving pain, beautifying the skin, preventing and treating cardiovascular and cerebrovascular diseases, possessing anti-bacterial and anti-tumor effects, etc. They have a wide range of pharmacological actions and clinical application values [7,8,9,13]. In addition, a patent technology showed that JBR had other pharmacological effects such as alleviating the side effects of chemotherapy, and was mainly used in food and healthcare [14]. KSR also has the effects of clearing heat and detoxifying, reducing inflammation and diuresis, and is used for rheumatic diseases and urinary tract infections. In the era of big health, the traditional Chinese medicinal value of PYR has been further explored, particularly with the joint publication of research findings on its antidepressant effects. This indicates that PYR has potential clinical application value in the field of mental health [4,15,16,17]. The main extraction method for rose essential oil is steam distillation, and its chemical components are complex, with the main active ingredients being olefin, alcohols, esters, ethers, aldehydes alkanes, etc. [3,6]. Due to the high complexity and variability of chemical components in traditional Chinese medicine, it is difficult to distinguish samples from different regions through a conventional direct comparison of chemical components [18]. However, up to now, no studies have been able to show the chemical differences among the volatile oils of the three types of roses. Therefore, based on the different functional roses circulating in the market, this research selected three types of roses (JBR, KSR, and PYR) as research subjects, attempting to identify and distinguish the varieties of roses from the components of rose essential oil.

Traditional comparative chemistry cannot identify the elements that cause quality differences. Chemometrics, based on computers and modern computational techniques, is a new interdisciplinary discipline [19]. In recent years, chemometric methods such as hierarchical cluster analysis (HCA), principal component analysis (PCA), partial least squares-discriminant analysis (PLS-DA), and orthogonal partial least squares-discriminant analysis (OPLS-DA) have been integrated with chemical analysis and have been widely applied in the identification, evaluation, and characterization of traditional medicines, foods, and fragrances [20,21,22,23]. HCA is a clustering technique that measures the differences or similarities between objects to be clustered. For classified groups of samples, HCA is frequently utilized, with the level of similarity being contingent upon the variable characteristics [24,25]. PCA is an unsupervised pattern recognition technique that uses linear dimensionality reduction to cluster and visualize high-dimensional data [26,27]. The supervised PLS-DA and OPLS-DA models are capable of more distinctly revealing the differences among compounds across various varieties [23,25]. Gas chromatography-mass spectrometry (GC-MS) is frequently used for the analysis and identification of volatile substances due to its high sensitivity, performance, and stability [28]. Therefore, this study used steam distillation to extract essential oils from the three types of roses and employed GC-MS for the qualitative analysis of volatile substances in essential oils from different rose varieties. The three types of rose essential oils were identified, differentiated, and aggregated using chemometric methods (including HCA, PCA, PLS-DA, and OPLS-DA). This article aims to investigate the chemical differences among the three rose essential oils, identify different rose varieties, and develop an effective strategy for the identification of rose varieties.

## 2. Results

### 2.1. Analysis of the Main Major Compound Classes in the Essential Oils of the Three Types of Roses

**Figure 1:**
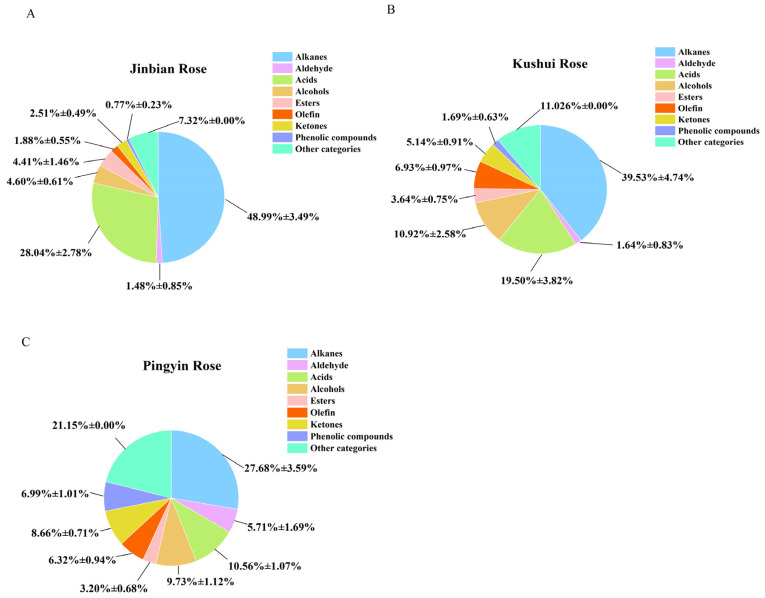
Pie chart of the content of the main components in the essential oils of the three types of roses. Jinbian Rose (**A**), Kushui Rose (**B**), Pingyin Rose (**C**).

Three varieties of dried roses (each with 10 batches) were analyzed, and their essential oils (30 batches in total) were characterized using GC-MS. All detected compounds were identified by matching against the NIST11.L and NIST14.L mass spectral databases, with structural information and relevant data successfully verified. In GC-MS analysis, the elution order of compounds generally follows the boiling point rule: lower-boiling-point compounds elute earlier (shorter retention times), while higher-boiling-point compounds elute later [26,27]. According to the total ion chromatography (TIC), the chemical components and contents among different batches of these three types of roses were similar, but there were still certain differences. JBR, KSR, and PYR were all dried roses with an average essential oil extraction rate of about 0.030% (Table 1). Table 2 and Figure 1 showed that the main chemical components in JBR essential oil were alkanes (48.99%), acids (28.04%), esters (4.41%), and alcohols (4.60%). In KSR essential oil, the main chemical components are alkanes (39.53%), acids (19.45%), alcohols (10.92%), and olefins (6.93%). In PYR essential oil, the main chemical components are alkanes (27.68%), acids (10.57%), alcohols (9.73%), and ketones (8.66%). The essential oils of JBR, KSR, and PYR all contain alkane, acid, and alcohol components, but the contents vary. In the essential oil of JBR, the combined content of alkanes, acids, and alcohols reached 81.63% (alkanes > acids > alcohols); in KSR essential oil, it reached 69.9% (alkanes > acids > alcohols); and in PYR essential oil, it reached 47.98% (alkanes > acids > alcohols). Among the essential oils of the three types of dried roses, the content trend of these three classes of components was similar, with alkanes having the highest content. In particular, the content of alkanes in JBR essential oil was as high as 48.99%, making it the variety with the highest alkane content among the three types of roses. Next were the acid components, in the JBR essential oil, the content of acid components was higher than in the other two types of rose essential oils. Alcohol components were the least abundant among the three types of components, with the highest content found in the KSR essential oil. In addition, the content of esters components in JBR essential oil (4.41%) was higher than in the other two types of rose essential oils. The content of olefin components in KSR essential oil (6.93%) was higher than in the other two types of rose essential oils, and the content of ketones in PYR essential oil (8.66%) was higher than in the other two types of rose essential oils.

### 2.2. Analysis of Specific Components in the Essential Oils of Three Types of Roses

The essential oils of JBR, KSR, and PYR were analyzed for their volatile components. In the JBR essential oil, 40 volatile components were identified, comprising 11 alkanes, 4 aldehydes, 12 acids, 6 alcohols, 4 esters, 1 ketone, 2 phenols, and 1 other compound (Table 2). For the KSR essential oil, a total of 48 volatile compounds were detected, including 10 alkanes, 5 aldehydes, 5 acids, 13 alcohols, 3 esters, 5 alkenes, 4 ketones, 2 phenols, and 7 other compounds (Table 2). In the case of PYR essential oil, 40 volatile compounds were identified, consisting of 10 alkanes, 1 aldehyde, 9 acids, 10 alcohols, 4 esters, 1 alkene, 1 ketone, 2 phenols, and 5 other compounds (Table 2).

### 2.3. Analysis of the Same Components in the Three Types of Dried Roses

Through the analysis of the main components in the essential oils of the three types of dried roses, it was found that there were eight similar components among the three roses (Table 2). The same eight components were furfural, heptacosane, tricosane, dodecanoic acid, heptacosane, phytol, tetradecanoic acid, and n-hexadecanoic acid. These eight components were present in every batch of the three types of dried roses, but their contents vary among each variety. Therefore, these eight compounds were selected as representative compounds to distinguish among the three types of roses. The cluster analysis was performed on three types of dried rose essential oils using HCA, PCA, PLS-DA, and OPLS-DA, with the content of these eight compounds as variables.

#### 2.3.1. Hierarchical Cluster Analysis (HCA)

**Figure 2:**
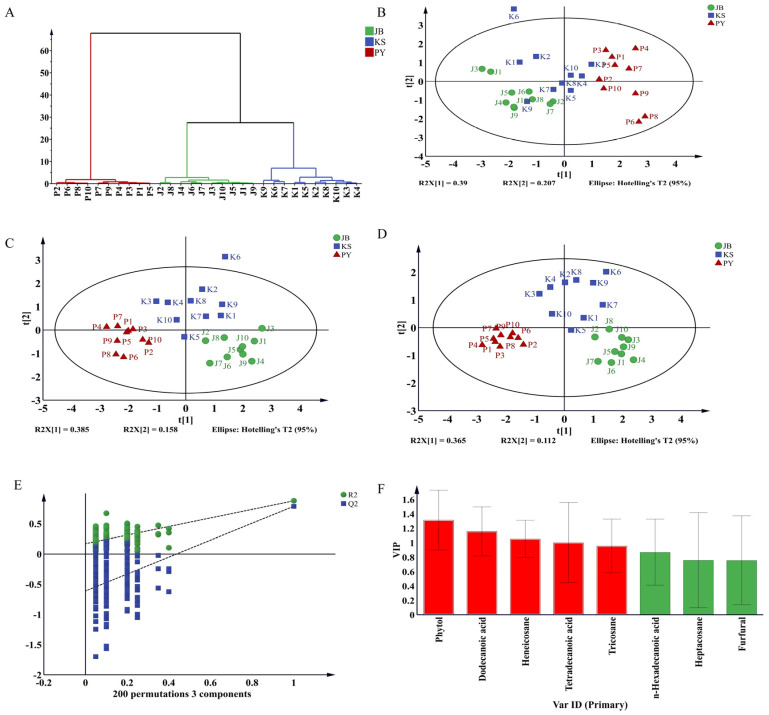
Cluster analysis diagram of the same eight components in three types of rose essential oils. HCA (**A**), PCA (**B**), PLS-DA (**C**), OPLS-DA (**D**), 200 times permutation test on the OPLS-DA of volatile components (**E**), plots of the variable importance in projection (**F**).

To further classify and distinguish the chemical components of the three types of dried rose essential oils, these eight common compounds were used as variables for the cluster analysis. Based on the relative content of each component in the essential oils, the HCA model roughly divided the roses into groups with similar characteristics in the same cluster (Figure 2A). The results indicated that the samples were divided into three groups, with the first group including J1–J10, the second group including K1–K2, and the third group including P1–P10. In other words, the three types of dried roses can be distinguished based on the components of their essential oils.

#### 2.3.2. Principal Component Analysis (PCA)

The PCA was conducted based on the eight characteristic components to provide more information on distinguishing the three types of dried roses. In the PCA plane model, the projection plot of the first two principal components is shown in Figure 2B. The 30 batches of rose essential oil samples were roughly divided into three groups, with PYR being well separated from the other two types of roses, but there was an overlap between the samples of JBR and KSR. The Q2 parameter of the PCA was <0.5, with R2X = 0.596, indicating that the model had poor predictive power and could not effectively distinguish between the three types of dried roses. Moreover, the K6 sample in the KSR deviated slightly from the confidence interval and might differ significantly from the other samples.

#### 2.3.3. Partial Least Squares-Discriminant Analysis (PLS-DA)

Although HCA and PCA can roughly distinguish the three types of dried roses, they fail to identify the variables responsible for sample classification. Therefore, the PLS-DA technique is used to visualize the differences among these samples [23]. Figure 2C was the PLS-DA score plot of the volatiles, with R2X = 0.762, R2Y = 0.849, and Q2 = 0.7, indicating that the discriminant model was reliable for predicting new data. In the PLS-DA plane model, the projection plot of the first two principal components is shown in Figure 2C. The 30 batches of rose essential oil samples were roughly divided into three groups, with samples of JBR, KSR, and PYR each clustering into their category, indicating that the three types of roses could be well distinguished from each other. However, as with PCA, there is a similar problem where the K6 sample from the Bitter Water Rose deviates slightly from the confidence interval, suggesting that it may be significantly different from the other samples.

#### 2.3.4. Orthogonal Partial Least Squares-Discriminant Analysis (OPLS-DA)

Although PLS-DA can effectively distinguish the three types of dried roses, the presence of an outlier in the model reduces its credibility. Therefore, to enhance the credibility of the model, the supervised OPLS-DA model was used [23,25]. The supervised OPLS-DA technique was used to analyze the relative content of eight characteristic components. The R2X, R2Y, and Q2 (cum) values of the OPLS-DA model were 0.853, 0.865, and 0.715, respectively, indicating that the model had good fitting and predictive capabilities. In the OPLS-DA plane model, the 30 batches of rose essential oil samples were successfully divided into three groups, with the three types of roses well clustered into one category (Figure 2D). It was worth noting that the K6 sample of KSR fell within the confidence interval, indicating that the model had high reliability.

In the permutation test, 200 substitutions were plotted for the OPLS-DA model, and the intercepts for R2Y and Q2Y were <0.4 and 0.05, indicating that the model was not overfitted and the model validation was effective (Figure 2E) [29]. The VIP (variable importance in projection) intuitively reflected the contribution of each variable of this OPLS-DA model classification and can be used as the most important original classification markers [30,31]. The five volatile compounds in Figure 2F were found to have VIP > 1, and can be considered potential indicators for the discrimination of the three types of dried roses. They were phytol, dodecanoic acid, heneicosane, tetradecanoic acid, and tricosane.

## 3. Discussion

### 3.1. Analysis and Comparison of the Main Components in the Essential Oils of Three Types of Roses

As shown in Table 2 and Figure 1, the three types of rose essential oils primarily consisted of nine compounds, though their respective compositions varied significantly. In JBR essential oil, the relative abundance of compound classes followed these orders: alkanes > acids > alcohols > esters > ketones > alkenes > aldehydes > phenols > benzenes; alkanes > acids > alcohols > alkenes > ketones > esters > phenols > aldehydes > benzenes in KSR essential oil; and alkanes > acids > alcohols > ketones > phenols > alkenes > aldehydes > esters > benzenes in PYR essential oil. All three types of rose essential oils contain high levels of alkane compounds, which are known for their anti-inflammatory and anti-oxidant properties [26]. These pharmacological effects are consistent with those of rose essential oil, suggesting that alkane compounds contribute significantly to its anti-oxidant and anti-inflammatory properties. Notably, the quantitative analysis revealed that acids and alcohols also represented the most abundant compound classes across all three rose varieties. The acids fraction of rose essential oil demonstrates significant bioactive properties, including anti-oxidant, anti-inflammatory, anti-tumor, and anti-bacterial effects [4]. Similarly, the alcohol components exhibit notable pharmacological activities such as anticholinesterase effects, anti-oxidant capacity, and bacteriostatic action [27]. Moreover, alcohols in rose essential oil are the main contributors to the fragrance of the oil, such as citronellol and geraniol. From an overall analysis, the compounds identified in this experiment are the primary active substances that make up rose essential oil, which is consistent with the literature reports.

### 3.2. Analysis of the Main Compounds in Three Types of Rose Essential Oils

The study identified 40, 48, and 40 volatile compounds in JBR, KSR, and PYR essential oils, respectively, with KSR essential oil containing the highest number of components. The results showed that the main chemical components detected in the essential oil of JBR were n-hexadecanoic acid (14.15% ± 0.14%), heneicosane (13.47% ± 0.12%), nonadecane (10.37% ± 0.16%), tricosone (6.12% ± 0.12%), etc. The main chemical components detected from the KSR essential oil were n-hexadecanoic acid (12.17% ± 0.54%), tricosone (11.79% ± 0.16%), heneicosane (8.55% ± 0.24%), 2-hexadecanol (2.47% ± 0.10%), etc. The main chemical components detected in the PYR essential oil were tricosone (12.87% ± 0.18%), heneicosane (9.20% ± 0.18%), methyleugenol (8.55% ± 0.21%), eicosane (9.20% ± 0.18%), etc. The identification results of these three rose essential oils are basically consistent with the literature reports, with only differences in content [7,32,33]. We speculate that this discrepancy may result from differences in sample preparation, as our study used dried rose specimens while previous research primarily employed fresh materials, potentially leading to variations in volatile compound composition. Table 2 indicated that the top eight compounds with the highest content in the essential oils of the three types of roses were very similar, with alkane compounds being the majority. It is worth noting that n-hexadecanoic acid was the highest in JBR and KSR and ranked third in PYR. Additionally, JBR had the greatest variety of alkane and acid substances. KSR had the greatest diversity of alkane and alcohol substances. In PYR, the diversity of alkanes and alcohols was greatest. In summary, by using the GC-MS technology to analyze the components in rose essential oil, it is possible to identify different varieties of rose essential oil from the perspective of their components.

A comparative analysis of the three rose essential oils revealed eight shared major constituents (Table 2). The cluster analysis was performed on three types of dried rose essential oils using HCA, PCA, PLS-DA, and OPLS-DA, with the content of these eight compounds as variables. Based on the results of HCA, PCA, PLS-DA, and OPLS-DA models, these eight components can effectively categorize the three types of roses into three distinct groups. Additionally, according to the VIP plot in Figure 2F, five volatile compounds (phytol, dodecanoic acid, heneicosane, tetradecanoic acid, and tricosane) were identified as potential indicators for the three types of roses. It is worth noting that these five compounds are among the higher content compounds in the three types of roses, and they also show great value in cluster analysis for distinguishing roses from different varieties. Phytol is a compound widely present in nature, with extensive pharmacological effects, including anti-inflammatory, anti-oxidant, anti-microbial, anti-tumor, and various other biological activities. Dodecanoic acid is a saturated fatty acid with various pharmacological effects, such as anti-inflammatory, anti-oxidant, and anti-tumor activities [34]. Heneicosane has potential applications in anti-bacterial, anti-fungal, and anti-oxidant areas, especially in the fields of medicine and biotechnology [35]. Tetradecanoic acid possesses pharmacological effects including anti-oxidant properties, promotion of energy metabolism, anti-inflammatory, anti-tumor, anti-hyperlipidemic, and anti-diabetic activities [36,37]. Tricosane, as an alkane, is primarily a component found in certain plant essential oils. The pharmacological effects of these compounds are similar to those of rose essential oil, and they can be used as chemical indicators to distinguish between different varieties of roses.

### 3.3. Future Prospects

Rose essential oil is renowned for its distinctive aromatic fragrance and possesses a variety of significant properties, such as sedative, anti-anxiety, and anti-oxidant effects [38,39,40]. These effects primarily originate from the intricate chemical composition of rose essential oil, which includes compounds such as linalool, citronellol, and dodecanoic acid. The synergistic action of these components bestows rose essential oil with a wide range of pharmacological properties [26]. Our research team has successfully incorporated rose (Kushui Rose and Pingyin Rose) essential oil into the formulation of novel nutraceutical products, demonstrating its potential for health applications. Although the pharmacological properties of rose essential oil are well-documented, the specific bioactive constituents responsible for these effects remain poorly characterized. To elucidate these active components, we conducted a comprehensive comparative analysis of essential oils derived from three distinct rose varieties. Our findings provide a detailed chemical profile of these essential oils, establishing a foundation for future structure–activity relationship studies. Subsequent research will focus on isolating and evaluating individual bioactive compounds to determine their specific pharmacological contributions, thereby enabling more targeted therapeutic applications of rose essential oil.

## 4. Materials and Methods

### 4.1. Materials

Dry roses purchased from three types of rose medicinal herbs (Chengdu Lotus Pond Traditional Chinese Medicine Professional Market, Chengdu, China) in the market on 5 October 2022 were used as experimental materials. The three types of roses are Shandong Pingyin Rose, Gansu Kushui Rose, and Yunnan Jinbian Rose, and 10 batches of each rose were purchased. All the rose samples used in this experiment comply with the Chinese Pharmacopoeia standards and have been dried and processed in accordance with national regulations.

### 4.2. Extraction Method of Rose Essential Oil

The rose volatile oil was obtained through hydrodistillation, following the method outlined in General Principles 2204 of the Chinese Pharmacopoeia. The distillation conditions are as follows: with water as an extraction solvent, 500 g of rose was added to a 10,000 mL round-bottomed flask in a material-to-liquid ratio of 1:8. At the same time, 200 mL of 5% NaCl solution (Chengdu Cologne Chemicals Co., Ltd., Chengdu, China) was added, and the rose herbs were soaked for 4 h before being used for extraction. After 4 h of hydrodistillation, the light yellow volatile oil was collected and stored at 4 °C in the dark. After drying with anhydrous sodium sulfate (Chengdu Cologne Chemicals Co., Ltd., Chengdu, China), the supernatant was dissolved in n-hexane (Chengdu Cologne Chemicals Co., Ltd., Chengdu, China) and passed through a 0.22 μm filter for GC-MS analysis.

### 4.3. Gas Chromatography-Mass Spectrometry (GC-MS)

Evaluation of the volatile components in rose was performed by using gas chromatography (7890A, Agilent, Santa Clara, CA, USA) coupled with mass spectrometry (5975C, Agilent, Santa Clara, CA, USA) with a 19091 N-133HP-INNOWax capillary column (30 m × 0.25 mm × 0.25 μm) (Agilent, Santa Clara, CA, USA). The injection volume was 1 µL with a 10:1 (*v*/*v*) ratio split mode. The carrier gas was helium (99.999% purity, 1 mL/min). The injector temperature was set at 230 °C and the detector temperature was 230 °C. The column temperature was initially held at 50 °C for 5 min, then increased 5 °C/min to 100 °C, and maintained at 100 °C for 5 min. Then, the column temperature was increased 8 °C/min to 140 °C and maintained at 140 °C for 6 min. Finally, the column temperature was increased 2 °C/min to 220 °C, and maintained at 220 °C for 10 min. The mass spectrometer was operated at an electron energy of 70 eV in full scan mode. The detected mass spectra were compared with those listed in the National Institute of Standards and Technology database (NIST11.L and NIST14.L), and the volatile components in rose were identified. The relative content of each component in the chromatogram was calculated based on the area normalization method [1,29,30,41].

### 4.4. Statistical Analyses and Software

All test samples were processed using the Ward method to visualize the differences and/or similarities among samples through an Euclidean distance. In the initial dataset, the relative contents of the eight characteristic compounds were treated as variables in the PCA model. The component distribution trends of rose samples from three different sampling regions were visualized by the principal component analysis (PCA), partial least squares-discriminant analysis (PLS-DA), and orthogonal partial least squares-discriminant analysis (OPLS-DA) models using SIMCA 14.1 software (Umetrics, Umea, Sweden). The bar charts and pie charts were drawn using Origin software 2021 (OriginLab Corporation, Northampton, MA, USA).

## 5. Conclusions

In this study, three commercially available common rose varieties were selected as research subjects and an effective strategy for rose variety identification was developed by combining the GC-MS analysis with chemometric methods and distinguishing and identifying the three types of roses from the perspective of essential oil components. The TIC chromatogram from the GC-MS analysis revealed that the essential oils of the three types of dried roses mainly consist of large molecular compounds, such as alkanes, acids, and esters. Additionally, some markers with significant identification value have been discovered. Based on the chemometric analysis (including HCA, PCA, PLS-DA, and OPLS-DA), it was found that there were differences in volatile components among the different types of roses. Moreover, it was found that eight characteristic components can effectively distinguish three types of dried roses, and samples from the same variety are clustered together. In summary, the combination of GC-MS analysis and chemometric methods can effectively distinguish different varieties of roses and serve as a reliable, robust, rapid, accurate, and low-cost analytical technique for evaluating the quality of rose essential oil. 

## Figures and Tables

**Table 1:** Three types of rose sample information and extracted essential oil content.

Rose Samples	Essential Oil/% (*v*/*w*)
Jinbian Rose	0.030 ± 0.006
Kushui Rose	0.028 ± 0.004
Pingyin Rose	0.032 ± 0.007

**Table 2:** The main components in the JBR, KSR, and PYR essential oils.

Compounds	Rt	Content %-Jinbian Rose	Content %-Kushui Rose	Content %-Pingyin Rose
Furfural	14.981	0.46 ± 0.05	0.44 ± 0.05	0.55 ± 0.08
Octadecane	15.542	/	/	0.49 ± 0.04
Benzaldehyde	16.750	0.30 ± 0.09	0.57 ± 0.10	/
Hexadecane	21.451	0.56 ± 0.05	0.83 ± 0.07	/
Levomenthol	21.604	/	/	0.54 ± 0.06
Benzeneacetaldehyde	21.613	1.33 ± 0.14	/	/
Aromandendrene	21.756	/	0.95 ± 0.04	/
Copaene	22.450	/	0.22 ± 0.02	/
trans-calamenene	22.905	/	2.44 ± 0.04	/
Pentacosane	22.945	/	/	6.02 ± 0.07 *
alpha-Terpineol	23.440	/	0.39 ± 0.04	0.14 ± 0.02
2-Dodecanol	24.192	/	1.02 ± 0.06	/
Heptadecane	24.296	0.50 ± 0.04	/	/
Dodecane	24.354	/	0.57 ± 0.05	/
Citronellol	25.105	0.85 ± 0.07	0.62 ± 0.05	0.65 ± 0.06
Acetic acid, 2-phenylmethyl ester	25.981	0.27 ± 0.03	/	0.44 ± 0.05
2-Tridecanone	26.162	0.49 ± 0.04	1.66 ± 0.18	0.63 ± 0.06
Tridecanal	26.438	0.52 ± 0.05	0.52 ± 0.09	/
Nonadecane	26.656	10.37 ± 0.16 *	/	/
Octacosane	27.751	0.35 ± 0.04	/	0.62 ± 0.06
Geraniol	27.018	/	0.56 ± 0.05	0.51 ± 0.06
Phenylethyl Alcohol	28.493	0.57 ± 0.05	0.70 ± 0.08	0.54 ± 0.05
2-Hexadecanol	29.540	/	2.47 ± 0.10 *	/
2-Tetradecanol	29.616	/	1.68 ± 0.08	/
Nonadecane	29.740	/	1.41 ± 0.05	1.22 ± 0.08
n-Pentadecanol	29.939	/	0.96 ± 0.08	0.510 ± 0.04
9-Nonadecene	30.101	/	0.46 ± 0.04	/
Methyleugenol	32.794	2.85 ± 0.08 *	0.82 ± 0.06	8.55 ± 0.21 *
Mandelic acid	33.279	0.31 ± 0.03	0.74 ± 0.05	/
2-Pentadecanone	33.517	/	/	/
Methoxyacetic acid, 2-tetradecyl	33.746	/	/	1.10 ± 0.07
Eicosane	33.936	3.36 ± 0.15 *	1.54 ± 0.09	/
Octanoic acid	33.420	1.55 ± 0.10	/	0.23 ± 0.03
Nerolidol 2	34.088	2.59 ± 0.10	/	/
Nerolidyl acetate	34.260	/	0.35 ± 0.04	/
Octanoic acid	34.545	/	0.92 ± 0.07	/
n-Tridecan-1-ol	35.373	0.33 ± 0.04	/	/
Cedrol	36.496	/	1.36 ± 0.08	/
1-Hexadecanol, acetate	36.591	/	/	1.61 ± 0.09
Pent-2-ynal	38.066	/	0.41 ± 0.08	/
Heneicosane	38.523	13.47 ± 0.12 *	8.55 ± 0.24 *	9.20 ± 0.18 *
Eugenol	38.761	0.77 ± 0.05	0.88 ± 0.08	3.54 ± 0.09 *
Nonanoic acid	39.236	1.21 ± 0.07	/	0.63 ± 0.04
1-Undecanol	39.817	/	/	0.44 ± 0.04
Isolongifolen-5-one	40.074	/	2.89 ± 0.05*	/
2-Benzyl-3-isopropyl-cyclopentanone	41.140	/	0.83 ± 0.06	/
alpha-Bisabolol	41.416	0.55 ± 0.05	1.89 ± 0.09	1.03 ± 0.05
Hexadecanoic acid, methyl ester	41.891	0.65 ± 0.06	/	/
Decanoic acid, methyl ester	42.091	/	0.47 ± 0.05	/
Hentriacontane	42.405	/	/	1.01 ± 0.05
Docosane	42.443	0.55 ± 0.06	/	/
Pentacosane	42.576	/	5.72 ± 0.21	/
n-Decanoic acid	43.623	1.03 ± 0.05	1.42 ± 0.08	1.08 ± 0.09
Citronellyl butyrate	45.050	/	2.81 ± 0.09	/
Isophytol	45.431	/	0.33 ± 0.08	/
Tricosyl acetate	45.450	/	/	0.425 ± 0.05
Tricosone	47.011	6.12 ± 0.12 *	11.79 ± 0.16 *	12.87 ± 0.18 *
1-Methoxymethyl-2-methylbenzene	47.981	1.78 ± 0.06	/	/
1,4-Pentadiene	47.991	/	2.79 ± 0.10 *	/
Benzenemethanol, 2,4-dimethyl-	48.295	/	/	0.62 ± 0.05
2,3-Dimethylanisole	48.486	/	1.52 ± 0.09	/
Tetracosane	50.893	/	/	1.07 ± 0.08
Isolongifolene, 9,10-dehydro-	52.264	/	/	1.42 ± 0.07
Dodecanoic acid	52.444	2.34 ± 0.10	2.83 ± 0.08 *	1.375 ± 0.11
trans-calamenene	52.463	/	1.56 ± 0.10	/
4-Isopropylcinnamic acid	52.930	/	/	0.56 ± 0.06
Eicosane	55.128	/	/	7.56 ± 0.18 *
Pentacosane	55.423	5.12 ± 0.10 *	/	/
3,4-Dimethylanisole	56.412		0.88 ± 0.09	/
Tridecanoic acid	56.812	4.83 ± 0.07	/	/
2-Aminoresorcinol	57.355	/	/	0.83 ± 0.06
Phytol	57.964	1.93 ± 0.04	2.19 ± 0.07	0.63 ± 0.05
Benzoic acid, 2-phenylmethyl ester	60.076	0.34 ± 0.03	/	/
Tetradecanoic acid	60.885	2.90 ± 0.08 *	0.55 ± 0.07	0.66 ± 0.05
cis, cis, cis-7,10,13-Hexadecatrienal	61.256	/	0.56 ± 0.05	/
Heptacosane	62.807	2.52 ± 0.10	2.374 ± 0.14	1.985 ± 0.13
Hexatriacontane	62.731		/	1.96 ± 0.10 *
Octacosyl trifluoroacetate	63.958	0.62 ± 0.03	/	/
Pentadecanoic acid	64.596	0.51 ± 0.04	/	/
n-Hexadecanoic acid	68.697	14.15 ± 0.14 *	12.17 ± 0.54 *	6.62 ± 0.23 *
Octadecanoic acid	76.500	0.54 ± 0.09	/	0.62 ± 0.05
Oleic Acid	77.737	0.96 ± 0.10	/	/
9,12-Octadecadienoic acid (Z, Z)-	80.84	3.44 ± 0.38	3.70 ± 0.10 *	0.58 ± 0.09

Note: The symbol “*” denotes the most abundant components in this type of rose. The symbol “/” denotes that this compound is not detected in the rose essential oil.

## Data Availability

The data presented in this study are available upon request from the corresponding author.
